# Determining Protein Secondary Structures in Heterogeneous Medium-Resolution Cryo-EM Images Using CryoSSESeg

**DOI:** 10.1021/acsomega.4c02608

**Published:** 2024-06-08

**Authors:** Salim Sazzed

**Affiliations:** Department of Computer Science, Old Dominion University, Norfolk, Virginia 23529, United States

## Abstract

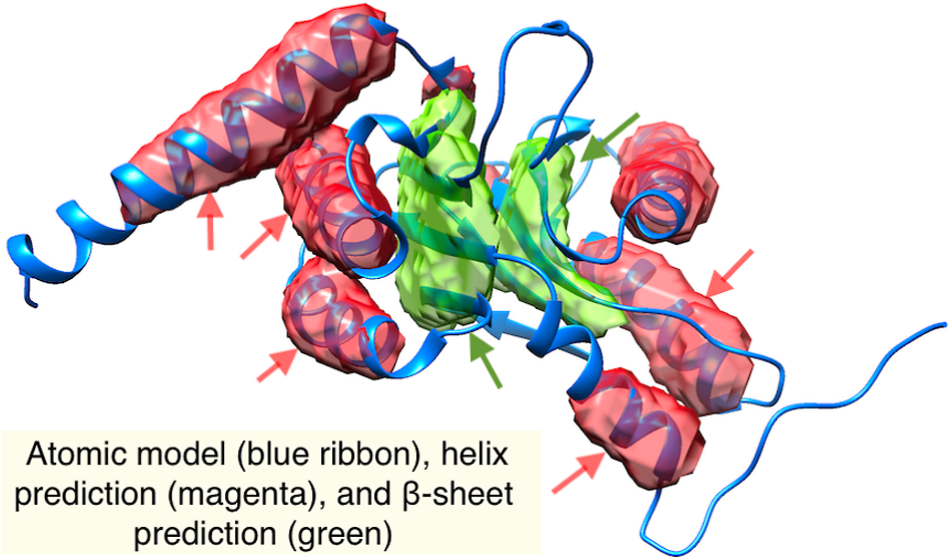

While the acquisition of cryo-electron microscopy (cryo-EM) at near-atomic resolution is becoming more prevalent, a considerable number of density maps are still resolved only at intermediate resolutions (5–10 Å). Due to the large variation in quality among these medium-resolution density maps, extracting structural information from them remains a challenging task. This study introduces a convolutional neural network (CNN)-based framework, cryoSSESeg, to determine the organization of protein secondary structure elements in medium-resolution cryo-EM images. CryoSSESeg is trained on approximately 1300 protein chains derived from around 500 experimental cryo-EM density maps of varied quality. It demonstrates strong performance with residue-level *F*_1_ scores of 0.76 for helix detection and 0.60 for β-sheet detection on average across a set of testing chains. In comparison to traditional image processing tools like SSETracer, which demand significant manual intervention and preprocessing steps, cryoSSESeg demonstrates comparable or superior performance. Additionally, it demonstrates competitive performance alongside another deep learning-based model, Emap2sec. Furthermore, this study underscores the importance of secondary structure quality, particularly adherence to expected shapes, in detection performance, emphasizing the necessity for careful evaluation of the data quality.

## 1 Introduction

Proteins serve a wide range of functionalities in living organisms, such as aiding in molecule transport, providing structural support, and boosting the immune system. They consist of a combination of 20 naturally occurring amino acids. The unique arrangements of these amino acids give rise to their distinct three-dimensional (3D) structures, ultimately determining their functions. Therefore, comprehending a protein’s 3D configuration is crucial for understanding its biological role.

Currently, one of the leading techniques for revealing the atomic arrangement of proteins is cryo-electron microscopy (cryo-EM). This method involves rapidly freezing samples in liquid-nitrogen-cooled liquid ethane and then imaging them with an electron microscope at extremely low temperatures.^1^ The increased accessibility of cryo-EM has greatly advanced the study of various molecules, including the generation of 3D density maps of proteins, also known as 3D density images, with resolutions primarily ranging from 3 to 20 Å^2^ and some achieving even better resolution. Density maps below a 5 Å resolution typically allow for the discernment of a protein chain’s backbone, enabling the derivation of near-atomic structures, although achieving high-accuracy structures typically necessitates a density map resolution closer to 3 Å. Conversely, when dealing with density maps above a 5 Å resolution (i.e., medium-resolution map), extracting the atomic structure directly becomes challenging due to the limited level of molecular details.

Protein secondary structures, such as α-helices and β-sheets, are the most distinguishable characteristics in a medium-resolution cryo-EM density map,^3^ even though amino acids are not discernible at such a resolution. In most medium-resolution maps, an α-helix resembles a cylinder, and a β-sheet appears as a thin layer of density in a medium-resolution map, although the general shape characters may be affected by their sizes and the density from the local environment of the molecule.

Over the years, various computational methods have been developed to derive atomic structures from density maps. The resolution of a cryo-EM density map is often considered an effective estimator for its quality. When the resolution reaches about 3.5 Å, density features become distinguishable for tracing the backbone of a protein chain.^4^ Phenix,^5^ a popular software for determining atomic structures, is initially developed for X-ray data and is subsequently extended for cryo-EM density maps of high resolution.^5^ For the medium-resolution density maps, a number of methods have been developed to detect secondary structure elements such as α-helices and β-sheets from cryo-EM density maps.^6−12^ These approaches rely on various algorithmic and classical image processing techniques. In practice, accurately detecting secondary structures poses a challenge due to the poor and nonuniform shape characteristics of a helix or a β-strand in medium-resolution images.^9,13−15^

Deep learning-based methods have demonstrated potential and performance across a spectrum of image segmentation tasks in diverse domains, including biological and medical fields in the early 2010s.^16,16−19^ Building on this inspiration, researchers introduced several approaches for protein secondary structure detection.^14,20^ For example, Li et al.^14^ proposed a deep learning-based model that utilizes a 3D CNN with inception learning and residual connections. The authors trained and tested their model using 25 simulated cryo-EM images. Similarly, Haslam et al.^20^ used a small data set primarily consisting of simulated data for protein secondary structure detection. The authors utilized 31 atomic protein structures from the Protein Data Bank (PDB), each simulated to a 9 Å resolution with a 1 Å voxel size. Out of these 31 3D images, 25 are allocated for training, while the remaining 6 are designated for testing purposes. Another related work is Emap2sec,^19^ which can also identify the secondary structures of proteins (α-helices, β-sheets, and other structures) in EM maps at resolutions of between 5 and 10 Å. Emap2sec utilizes patch-based cropping to retrieve secondary structure elements.

In this work, we present a U-net-based segmentation model, cryoSSESeg, for detecting protein secondary structures in medium-resolution cryo-EM 3D images. CryoSSESeg utilizes masked protein chain images generated by isolating individual protein chains from the protein-level atomic model. Afterward, it utilizes the coordinate information on these chains to extract and mask the corresponding regions in the density map. Employing chain-based cropping ensures the presence of entire secondary structures in the image, facilitating more effective learning of the model. Nonetheless, it is imperative to acknowledge that this approach may incur high memory requirements, especially when processing large protein chains. For labeling individual voxels in the density map, we utilize the STRIDE secondary structure annotation tool.^21^ The network is trained using 1268 protein chains isolated from approximately 500 cryo-EM density maps. To address the class-imbalance issue, we explore various types of loss functions, including weighted cross-entropy and dice loss. Results from a test set comprising 33 cryo-EM density maps exhibit an overall *F*_1_ score of 0.76 for helix detection and 0.60 for β-sheet detection at the residue level.

## 2 Materials and Method

### 2.1 Data Quality Evaluation and Selection

The cryo-EM density maps are downloaded from the Electron Microscopy Data Bank (EMDB)^a^ having a resolution between 5 and 10 Å and a corresponding atomic structure available in the PDB. The atomic structures of individual protein chains are utilized as envelopes to extract the bounding density box of the chains, which results in a set of model and map pairs, where each represents the atomic model and the corresponding density map of a chain. Given the common occurrence of multiple copies of the same chain in a cryo-EM density map, duplicate and nearly identical copies are removed to mitigate the training bias. The Needleman–Wunsch algorithm is employed to determine very identical and duplicate chains (i.e., those with more than 70% shared sequence identity) and is then excluded. Importantly, no chain in the testing data shares more than 35% sequence identity with any chain in the training set.

**Figure 1:**
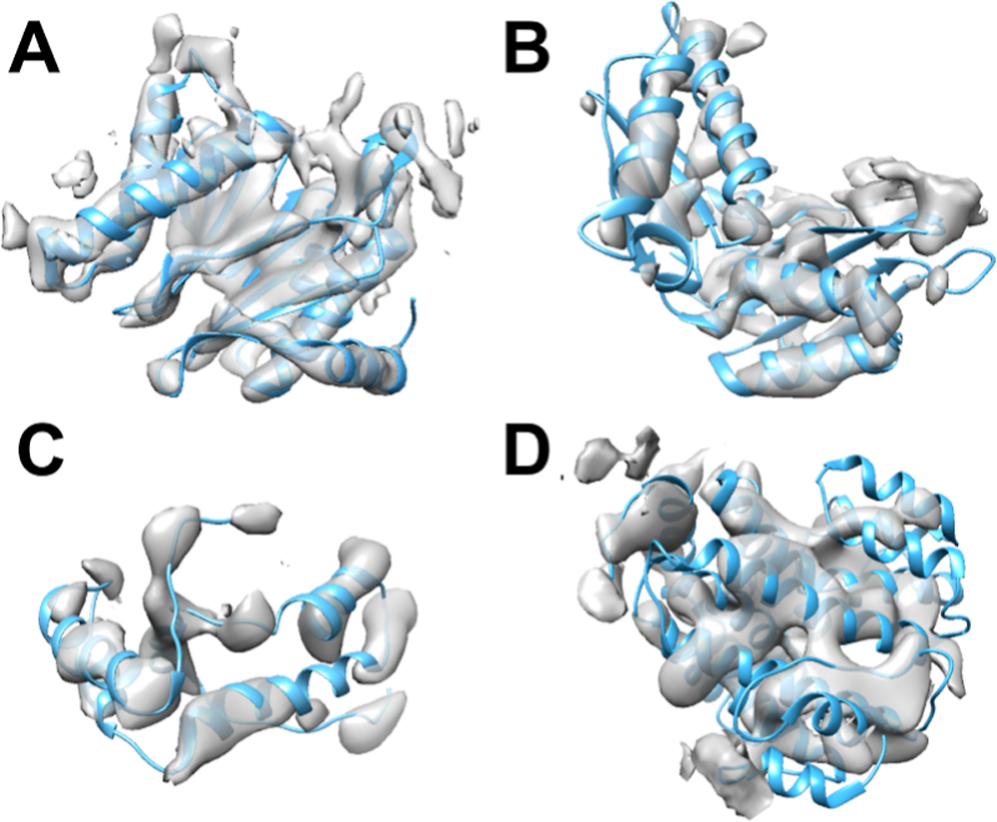
Cryo-EM density maps with variable map-to-model agreements at the secondary structure level. (A) Chain H (EMDB ID: 1733, 6.8 Å resolution) from Bin 1, (B) chain C (EMDB ID: 4075, 5.35 Å resolution) from Bin 2, (C) chain Q ( EMDB ID: 8129, 7.80 Å resolution) from Bin 3, and (D) chain W (EMDB ID: 6286, 8.30 Å resolution) from Bin 4. Atomic models are shown as ribbons.

It is known that the quality of density maps varies greatly in medium resolution, affecting the agreement between the density map and the atomic structures, especially concerning the protein secondary structure.^22^ To exclude low-quality data during training, we conducted a screening process, discarding chains with very poor matches between their atomic structures and density maps at the secondary structure level, particularly with respect to helix. The quality of each extracted density map (of a chain) is evaluated by its cylindrical fit of helices.^22^ More specifically, an *F*_1_ score is calculated from the fit for each helix in a chain, and the average *F*_1_ score over all helices is subsequently computed and used to score the overall quality of the density map. We observed mean and standard deviation scores of 0.55 and 0.1, respectively, across 4935 chains from around 500 proteins. These scores are then used to create four bins, roughly representing chains with varying qualities (shown in Figure 1). Bin 1 is the group of protein chains with the highest scores, indicating the best-estimated agreement between the helices in the models and the expected cylindrical shapes in the corresponding locations in the density maps. Each chain in Bin 1 has a score of at least 0.7, which is 1.5 standard deviations above the overall mean. It is observed that a larger difference between precision and recall resulting from the cylindrical fit (check ref (22) for details) often suggests a worse agreement between atomic structure and its density map. Therefore, to create Bins 2, 3, and 4, which contain chains with progressively deteriorating quality, the difference between precision and recall scores is also considered in addition to the overall quality score. Finally, Bin 2 contains protein chains with a score between 0.6 and 0.7, having a precision–recall difference below 0.15. Bin 3 contains protein chains with a quality score between 0.6 and 0.7 and a precision–recall difference of above 0.15. It also contains protein chains with a quality score between 0.55 and 0.6, with a stricter restriction on a precision–recall difference below 0.15. Bin 4 contains all remaining protein chains in the data set.

Since the chain representing Bin 4 hardly resembles the expected shape and quality of the protein secondary structures, it is excluded from this study. Only chains from the first three bins are used for training and evaluation. Additionally, we apply constraints related to the size of the chains. The chain images can vary in size, with typical chain sizes ranging from 16 to 100 Å in any of the *x*, *y*, and *z* directions. However, there may be some chains with higher dimensions along any axis. Considering the typical size of the chain and the memory required to process individual chains in RAM, we set a maximum limit of 100 voxels in any direction. Finally, a set of 1350 chains, including 238, 612, and 500 chains from bins 1, 2, and 3, respectively, is obtained after applying this size-based constraint. The total 1350 chains are partitioned into three disjoint subsets: training (*N* = 1268), testing (*N* = 33), and validation (*N* = 49). The splitting of training, testing, and validation is performed in such a way that it ensures a similar presence of protein chains representing different bins in these three sets. Each density map is resampled to 1 Å per voxel to make the annotation and training process uniform. The STRIDE server is used to annotate secondary structures. In the STRIDE annotation, H, G, and I are considered as the helix residues, while B, b, and E are considered as β-sheet residues. Voxels within 3 Å from the Cα atom of the helix and β-sheet residues are labeled as helix voxel and β-sheet voxels, respectively. In case a voxel has both helix and β-sheet Cα atom within a 3 Å radius, the helix has higher precedence. The rest of the voxels are labeled as background.

### 2.2 Architecture

CryoSSESeg is designed to process the entire chain image instead of splitting it into multiple fixed-size patches. A fixed patch size likely cuts secondary structures at any position, which may impact the secondary structure shape information available in the image. The network for protein secondary structure detection is adapted from the 3D U-Net model, proposed in ref (23). Given that the training set is relatively small, to prevent overfitting and reduce training time, five layers with dropout integrated are used. Moreover, to capture more contextual information and complex patterns, the number of feature channels is doubled in each convolution.

**Figure 2:**
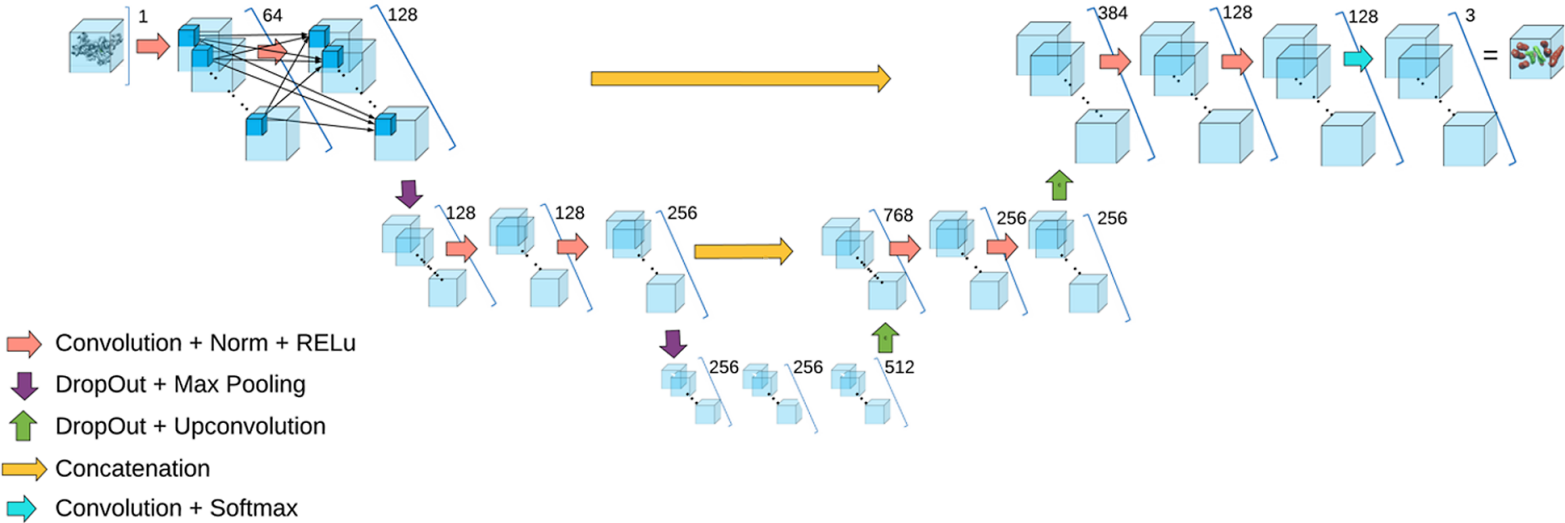
Architecture of the adapted 3D U-Net. Each blue block represents the activation map resulted from the operations as indicated.

Similar to other U-Net-based architectures, the network comprises a down-sampling path (also known as a contracting path), which includes the first two layers followed by the bottleneck layer, and an up-sampling path that includes the last two layers (Figure 2). The third layer (right in the middle) is the bottleneck layer, positioned between the contracting and expansive paths, which further compresses spatial information and enhances feature representation by increasing the number of channels. Each layer in the network consists of two convolutions using 3 × 3 × 3 kernels (or filters), each followed by a batch normalization and a ReLu nonlinearity. Each layer in the down-sampling path ends with a 2 × 2 × 2 max pooling with a stride of two, reducing the resolution by half in all dimensions. In contrast, each layer in the up-sampling path begins with a 2 × 2 × 2 transpose convolution with a stride of two to bring up the resolution by a factor of 2. Dropout is added to each layer right before the max-pooling in the down-sampling path or transpose convolution in the up-sampling path. In the last layer, a 1 × 1 × 1 convolution is used to decrease the number of output channels to three, corresponding to the three classes, i.e., helix, β-sheet, and background, respectively. The network contains 6,142,723 total trainable parameters. The maximum receptive field in the network is 35 × 35 × 35 voxels.

There is a significant class imbalance in the 3D cryo-EM image data used in this work, particularly between the foreground (helices and β-sheets) and background voxels (Table 1). One of the popular loss functions, cross-entropy, is known to have a problem with class imbalance. Specifically, models trained using cross-entropy often exhibit poorer performance on voxels belonging to classes with fewer instances, despite achieving a relatively high overall accuracy. In contrast, the dice coefficient is less affected by class imbalance, particularly between foreground and background voxels. However, it is less smooth, making it difficult to optimize. To address this class imbalance, cryoSSESeg utilizes a combination of weighted cross-entropy and the dice coefficient. For the weighted cross-entropy, the weights assigned to classes are set in inverse proportion to their presence in the image.

**Table 1:** Distribution of Voxels Based on Class-Labels (Out of 1)^a^

data set	background	helix	sheet
overall	0.992	0.004	0.003
training	0.992	0.004	0.003
validation	0.992	0.005	0.003
testing	0.992	0.004	0.004

a The background voxels make up almost all voxels in the density maps.

To prevent overfitting, kernel regularization is incorporated in addition to dropout. CryoSSESeg is implemented using the TensorFlow API. The Adam optimizer^24^ is utilized for optimizing the network weights. To determine hyperparameters, an extensive search is conducted with the aid of validation data. The chosen values are as follows: dropout rate of 0.5, learning rate of 0.001, batch size of 1, and 40 epochs.

## 3 Results and Discussion

### 3.1 Performance Evaluation

To assess the efficacy of cryoSSESeg, we consider both the voxel-wise and residue-specific *F*_1_ scores. The voxel-wise *F*_1_ score quantifies the agreement between the framework’s predictions for each voxel and the corresponding ground truth labels. Here, precision and recall are calculated for both helical and sheet secondary structures based on the counts of true-positive, false-positive, and false-negative voxels. The *F*_1_ score is then computed using these precision and recall values.

The residue-specific *F*_1_ score is calculated by comparing the predictions and true labels at the amino acid level. For each Cα atom (of the amino acid) present in the atomic model, its class prediction is determined by counting the class of individual voxels within a 3 Å radius, and then, the class with the maximum votes is assigned as the prediction. These class predictions of Cα atoms are then compared with the corresponding true labels obtained through STRIDE, a protein secondary structure annotation tool. Finally, precision, recall, and *F*_1_ scores are computed similarly to voxel-wise evaluations.

### 3.2 Evaluation on Varied-Quality Data

**Figure 3:**
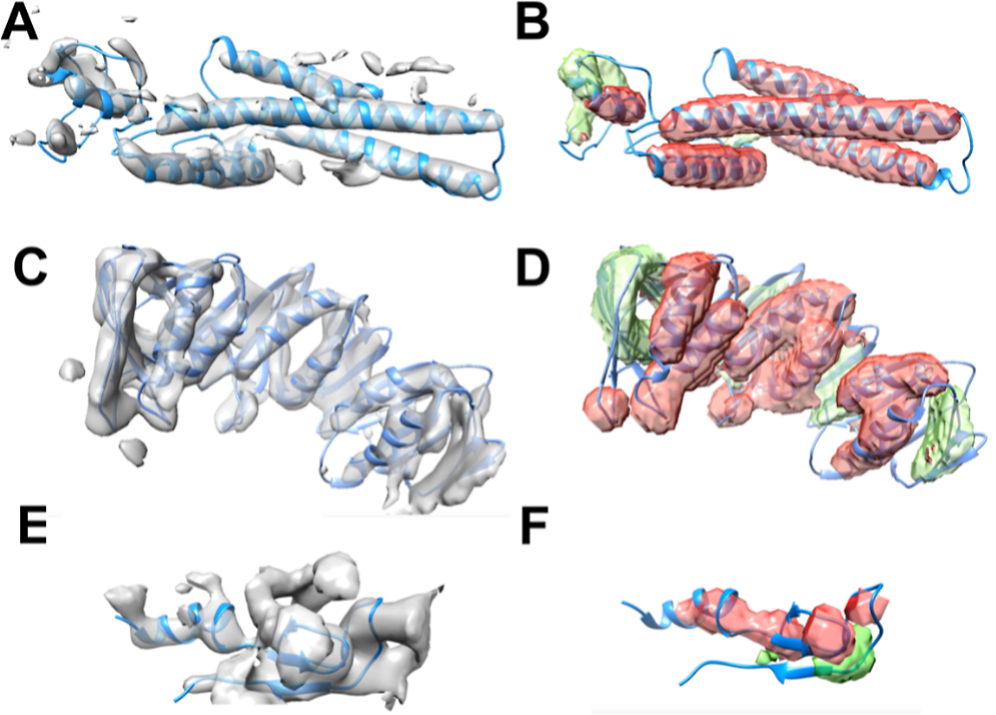
CryoSSESeg’s predictions for three protein chains exhibit varying levels of accuracy: the top panel (protein chain: 8518_5u8s_A, highly accurate), the middle panel (protein chain: 4141_5m1s_B, moderately accurate), and the bottom panel (protein chain: 5943_8j6y_80, low accuracy). Panels A, C, and E display the STRIDE annotated models alongside density maps, while panels B, D, and F illustrate predictions by cryoSSESeg (red indicates helix prediction, and green indicates β-sheet prediction) with the STRIDE annotated models.

The performance of cryoSSESeg is evaluated using 33 test cases: 8, 13, and 12 protein chains from Bin 1, Bin 2, and Bin 3, respectively (Table 3). Three examples of secondary structures segmented from cryo-EM density maps are presented (Figure 3), each demonstrating varying levels of detection accuracy. In the first example (top row of Figure 3), a high-quality cryo-EM density map (EMDB-8518) from Bin 1 that corresponds to chain A of PDB-5u8s is shown. This chain comprises 114 Cα atoms within helices and 13 Cα atoms within the β-sheet (see Table 2). CryoSSESeg successfully identifies all helices and a segment of the β-sheet region, achieving voxel-level *F*_1_ scores of 0.78 and 0.55 for helix and β-sheet detection, respectively (refer to Table 2). The second example (second row of Figure 3) is from Bin 2 and contains a much larger chain (4141_5*m*1*s*_*B*) comprising 366 amino acids, with 158 of them forming β-sheets (see Table 2). CryoSSESeg yields voxel-level *F*_1_ scores of 0.52 and 0.50 for helix and β-sheet detection, respectively. These two cases represent distinct bins based on cylindrical fits for the helices. In the first example, longer helices exhibit a more uniform cylindrical nature (see Figure 3). The better recognition of β-sheets in the second example may be attributed to larger β-sheet structures, highlighting the challenge of accurately detecting smaller β-sheets, such as those comprising 13 amino acids. It is important to note that bin partitioning is solely based on helix properties.

**Table 2:** *F*_1_ Scores for Helix and β-Sheet Detection across 33 Testing Protein Chains

EMDB_PDB_chain (resolution Å)	bin	Cα (H/S/T)	*F*_1_ voxel		*F*_1_ residue	
			helix	sheet	helix	sheet
1657_4*v*5*h*_*AE*(5.80)	1	42/38/150	0.60	0.50	0.69	0.58
1798_4*v*5*m*_*AE*(7.80)	3	42/58/150	0.65	0.51	0.81	0.69
2994_5*a*21_*G*(7.20)	3	37/21/133	0.58	0.53	0.75	0.53
3206_5*fl*2_*K*(6.20)	3	12/47/106	0.40	0.45	0.39	0.64
3491_5*mdx*_*H*(5.30)	2	33/0/42	0.71	NA	0.83	NA
3594_5*n*61_*E*(5.80)	3	86/52/212	0.61	0.54	0.71	0.68
3850_5*oqm*_4(5.80)	2	128/49/297	0.61	0.63	0.71	0.72
3850_5*oqm*_*g*(5.80)	1	81/0/85	0.75	NA	0.96	NA
3948_6*esg*_*B*(5.40)	2	51/0/78	0.74	NA	0.90	NA
4041_5*ldx*_*H*(5.60)	1	199/0/296	0.73	NA	0.89	NA
4041_5*ldx*_*I*(5.60)	2	48/19/176	0.52	0.40	0.64	0.57
4078_5*lms*_*D*(5.10)	2	86/18/208	0.63	0.37	0.72	0.40
4107_5*luf*_*M*(9.10)	3	322/0/439	0.53	NA	0.70	NA
4141_5*m*1*s*_*B*(6.70)	2	81/158/366	0.52	0.50	0.56	0.64
4182_6*f*42_*G*(5.50)	3	16/66/180	0.37	0.40	0.43	0.61
5036_4*v*69_*AD*(6.70)	3	78/19/205	0.58	0.34	0.68	0.38
5942_3*j*6*x*_25(6.10)	3	25/5/70	0.48	0.00	0.58	0.00
5943_3*j*6*y*_80(6.10)	3	12/8/52	0.35	0.10	0.53	0.22
6149_3*j*8*g*_*W*(5.00)	3	19/48/94	0.53	0.67	0.72	0.89
6446_3*jbi*_*V*(8.50)	2	116/0/131	0.74	NA	0.95	NA
6456_3*jbn*_*AL*(4.70)	3	89/8/211	0.69	0.38	0.82	0.30
6810_5*y*5*x*_*H*(5.00)	2	38/10/100	0.53	0.56	0.78	0.58
7454_6*d*84_*S*(6.72)	3	65/34/142	0.51	0.45	0.65	0.55
8016_5*gar*_*O*(6.40)	2	65/0/80	0.64	NA	0.81	NA
8128_5*j*7*y*_*K*(6.70)	1	71/0/93	0.78	NA	0.88	NA
8129_5*j*8*k*_*AA*(7.80)	2	187/60/446	0.59	0.41	0.71	0.50
8129_5*j*8*k*_*D*(7.80)	2	170/41/384	0.60	0.29	0.70	0.40
8130_5*j*4*z*_*B*(5.80)	1	63/9/154	0.67	0.44	0.75	0.42
8135_5*iya*_*E*(5.40)	2	88/44/210	0.65	0.60	0.78	0.70
8357_5*t*4*o*_*L*(6.90)	1	106/0/160	0.69	NA	0.83	NA
8518_5*u*8*s*_*A*(6.10)	1	114/13/208	0.78	0.55	0.94	0.46
8693_5*viy*_*A*(6.20)	1	68/0/133	0.67	NA	0.75	NA
9534_5*gpn*_*A*(5.40)	2	59/0/88	0.71	NA	0.89	NA
weighted average	ALL	2697/825/5879	0.63	0.48	0.76	0.60

**Table 3:** Average *F*_1_ Scores for Helix Detection across Bins

chain quality (based on helix)	num of chains	avg. *F*_1_	
		voxel	residue
Bin 1	8	0.71	0.84
Bin 2	13	0.63	0.77
Bin 3	12	0.52	0.65

Among the 33 test cases, the third example (chain 80 of PDB entry 3j6y), which represents Bin 3, exhibits the lowest performance. It consists of 12 helix residues and 8 β-sheet residues, totaling 52 amino acids. In this case, the residue-level *F*_1_ scores for helix and β-sheet detection are observed as 0.53 and 0.22, respectively (see Table 2). Upon visual inspection, we notice that the helix density lacks the distinct cylindrical shape observed in the density maps representing higher bins. Similarly, the β-sheet region lacks the typical thin surface characteristic. Although the entire cryo-EM map (EMD-5943) boasts a decent resolution of 6.1 Å, indicating good overall image quality, it fails to capture the shape characteristics of local regions, such as secondary structure elements.

While it is commonly assumed that the performance of secondary structure detection is influenced by the quality of the density map, there has been limited quantitative research on this aspect. In addition to determining protein secondary structure locations, we also analyze how the prediction performance is affected by the quality of the 33 test cases, computed through cylindrical fit scores and representing particular bins. We observe that the pattern of the average *F*_1_ scores is linked to the estimated bins. The average voxel-level *F*_1_ score decreases progressively from 0.71 in Bin 1 to 0.63 in Bin 2 and ultimately to 0.52 in Bin 3, attributed to a reduction in the cylindrical fit estimation. Interestingly, despite Bin 3 dominating over Bin 1 and Bin 2 in the training data set, helix detection performance is still the weakest in Bin 3. The density patterns of helices in Bin 3 are irregular and less consistent, as observed by the low cylindrical fit, which means a lower level of agreement between an atomic model and density in the helix region for Bin 3, likely responsible for that.

### 3.3 Comparison of Performance between CryoSSESeg and SSETracer

SSETracer is a well-known algorithmic method for detecting helices and β-sheets in medium-resolution cryo-EM density maps.^9^ It is publicly available as a plugin to Chimera,^25^ a popular visualization tool for cryo-EM density data and atomic structures. SSETracer relies on a set of topology-based algorithms such as skeletonization, local structural tensor, local thickness, and density values. The skeleton is derived from the density map using Gorgon.^26^ While SSETracer can yield good accuracy, its primary challenges include the dependency on a third-party tool, Gorgon,^26^ for skeleton generation, and the sensitivity of the results to user-specified parameters, a common issue in many image-processing-based methods. One of the important steps is the density threshold. To examine the effect of density thresholds, 20 equally spaced values between the mean and the 5 standard deviations of the density map are considered. An automation script has been developed to generate the skeleton for each sample threshold from Gorgon using Autohotkey (https://www.autohotkey.com) since Gorgon is a user-interactive program. The automation script executes SSETracer using the same sample threshold used for deriving the skeleton. The residue-level *F*_1_ scores are calculated for the detected helix and β-sheet voxels for each of the sample thresholds.

The examination of *F*_1_ scores using 20 density thresholds shows that the density threshold is an important parameter affecting the detection accuracy of SSETracer. The threshold needed to detect helices is generally higher than that for the detection of β-sheets. This is because helices are generally denser regions than β-sheets. Although SSETracer has the potential to detect secondary structures quite accurately, it is not trivial in practice for a nonexperienced user due to the choice of a density threshold. For each test case, the best of the 20 helix *F*_1_ scores is examined (column 5 of Table 4), as well as the average of three nonzero *F*_1_ scores (BestNbr *F*_1_ score, column 7 of Table 4) that are obtained from three consecutive thresholds near the one producing the highest *F*_1_ score. For the 12 tested cases, the average best *F*_1_ score reduced from 0.81 to 0.73 for helix detection and from 0.58 to 0.47 for β-sheet detection if an additional two consecutive density thresholds near the best threshold are considered. This experiment shows that SSETracer is sensitive to the change of about 25% of a standard deviation in density threshold for some cases, even near the best threshold. It is also generally not advisable to expect a universal best threshold for all test cases since a specific threshold needs to be considered for each test case. This is shown in our experiment of using the same threshold for all test cases, such as 3.25 and 2.5 standard deviations (columns 9 and 10 and columns 11 and 12 in Table 4) of each density map, and the average *F*_1_ score for the 12 cases is greatly reduced.

**Table 4:** Comparison of Residue-Level *F*_1_ Scores of CryoSSESeg and SSETracer^a^

EMDB_PDB_chain (resolution)	Cα	cryoSSESeg		SSETracer							
				Best		BestNbr		Std-3.25		Std-2.5	
	(H/S/T)	H	S	H	S	H	S	H	S	H	S
5036_4v69_AD (6.70)	78/19/205	0.68	0.38	0.71	0.34	0.69	0.21	0.63	0	0.72	0.09
5942_3j6x_25 (6.10)	25/5/70	0.58	0.00	0.8	0.29	0.67	0.19	0.8	0	0.61	0
5943_3j6y_80 (6.10)	12/8/52	0.53	0.22	0.93	0.61	0.67	0.30	0.52	0	0.58	0.62
6149_3j8g_W (5.0)	19/48/94	0.72	0.89	0.70	0.75	0.58	0.66	0.71	0	0.33	0.48
6446_3jbi_V (8.50)	116/0/131	0.95	NA	0.93	NA	0.91	NA	0.92	NA	0.82	NA
6810_5y5x_H (5.00)	38/10/100	0.78	0.58	0.76	0.63	0.73	0.26	0.76	0	0.46	0.64
7454_6d84_S (6.72)	65/34/142	0.65	0.55	0.76	0.69	0.55	0.54	0.48	0.45	0.25	0.46
8016_5gar_O (6.40)	65/0/80	0.81	NA	0.94	NA	0.82	NA	0.83	NA	0.7	NA
8128_5j7y_K (6.70)	71/0/93	0.88	NA	0.95	NA	0.94	NA	0.93	NA	0.94	NA
8130_5j4z_B (5.80)	63/9/154	0.75	0.42	0.79	0.63	0.61	0.40	0.74	0	0.79	0.64
8135_5iya_E (5.40)	88/44/210	0.78	0.70	0.68	0.45	0.67	0.43	0.66	0.43	0.33	0.45
9534_5gpn_Ae (5.40)	59/0/88	0.89	NA	0.77	NA	0.63	NA	0.78	NA	0.42	NA
**weighted average**		0.79	0.62	0.81	0.58	0.73	0.47	0.75	0.19	0.61	0.43

a The number of Cα atoms are shown forhelix (H), β-sheet (S), and total (T) for different chains. For each chain, SSETracer (Best): the highest *F*_1_ score obtained considering 20 samples of density threshold, SSETracer (BestNbr): the average of three *F*_1_ scores obtained using three density thresholds in the neighborhood of the one that produced the best *F*_1_ score, SSETracer (Std-3.25) and SSETracer (Std-2.5): the *F*_1_ scores obtained using a density threshold of 3.25 standard deviation and 2.5 standard deviation of the density map, respectively.

Results from the 12 test cases show that the efficacy of cryoSSESeg is very similar to the best performance of SSETracer for helix detection without manually selecting and sampling many different thresholds. Without knowledge of the ground truth in practice, the best performance when SSETracer is applied is unknown. The main advantage of cryoSSESeg is that it offers robust performance without requiring users to choose various parameters. Additionally, it is observed that cryoSSESeg demonstrates a much better *F*_1_ score than SSETracer for β-sheet detection, with an average *F*_1_ score of 0.62 compared to SSETracer’s 0.58. β-Sheets are generally more challenging to detect accurately than helices due to their lower density and dynamic shapes.

### 3.4 Comparison with Emap2sec

The design of cryoSSESeg and Emap2sec shares both similarities and differences. Both methods utilize CNN-based architectures to train the model for secondary structure detection in medium-resolution cryo-EM density maps. Emap2sec architecture is specifically designed for training on cryo-EM maps at the protein level.^19^ To address the substantial memory requirements, the authors extracted small patches of 11 Å cube size from the density maps and utilized them as inputs. While this approach effectively reduces memory usage, it may also lead to the loss of secondary structure information, resulting in incomplete or partial data within individual patches. Additionally, they implemented density-based thresholding, considering only voxels with density values higher than a specified threshold recommended by the depositors of the corresponding density map.

In contrast, cryoSSESeg is designed for individual chain components that are often much smaller than the entire cryo-EM map. Instead of using patches, the input to the network allows density maps of different sizes from 16 to 100 Å in any of the *x*, *y*, and *z* directions. Using the entire density map of a protein chain eliminates artifacts of chopping secondary structures. Although the training limits density maps to within 100 Å in any dimension, the limit does not apply to any dimension in testing. Emap2sec uses two phases in the architecture, while cryoSSESeg uses single phase.

**Figure 4:**
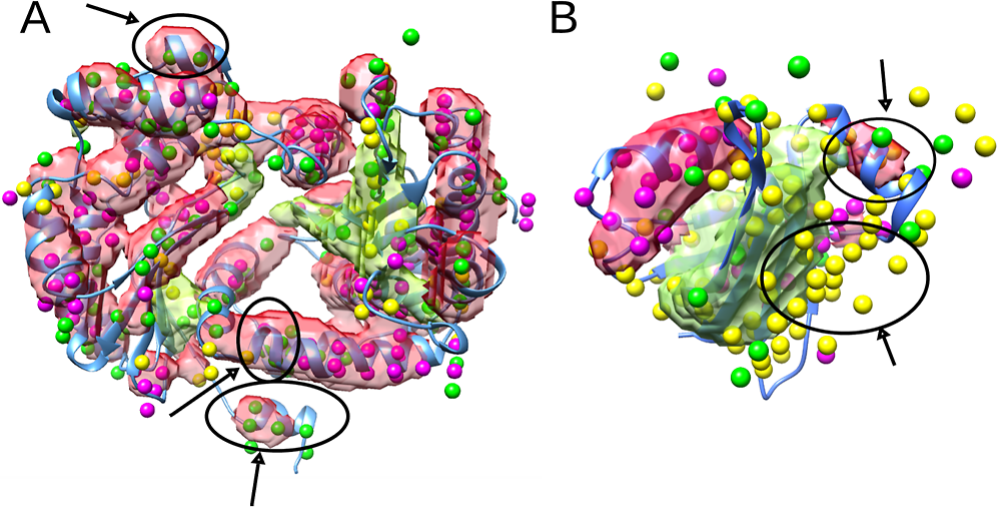
Two examples of cryo-EM density maps, EMD_8129_5j8k_AA in (A) and EMD_6149_3j8g_W in (B), are used to compare the segmentation results of cryoSSESeg and Emap2sec. The segmented helix volume (transparent red surface) and β-sheet volume (transparent green surface) are detected using cryoSSESeg. The detected helix voxels (purple dots), β-sheet voxels (yellow dots), and other voxels (green dots) are obtained using Emap2sec. Some differences are shown in circles and arrows.

Regardless of differences in the design, both methods are capable of detecting the overall regions of most helices and β-sheets. For Emap2sec, publicly available code and data from Code Ocean (https://codeocean.com/capsule/4873360/tree) are used. A standard procedure is used according to the tutorial, with stride size = 2, vm = 5, and a density contour suggested by the density map. CryoSSESeg uses 1 Å per voxel to represent the segmented volume. It is observed that the predictions (output voxel) of Emap2sec are sparse, and our attempt to use finer sampling points failed. In the examples shown (Figure 4), it is observed that the prediction of helixes (red surface and purple dots) mostly overlap for longer helices but have some differences in some shorter ones (circles in Figure 4). Using detected helix voxels and β-sheet voxels, residue-level accuracy is calculated for helix Cα atoms and β-sheet Cα atoms. As an example, a helix Cα atom is determined using maximum voting of voxel labels within 3 Å-radius from the Cα. For the case *EMD*_6149_3*j*8*g*_*W* (Figure 4 B), the residue-level helix accuracy is 0.47 and 0.21 for cryoSSESeg and Emap2sec, respectively. The difference in accuracy may reflect the missed helix in the detection and sparseness of voxels produced using Emap2sec. However, the current implementation of both methods limits a more rigorous comparison.

## 4 Summary and Conclusions

The relative positions of protein secondary structures provide crucial constraints for deriving the atomic structure of a protein. In this study, we present a 3D convolutional neural network (CNN)-based framework, cryoSSESeg, for segmenting secondary structures, such as helix and β-sheets, in medium-resolution cryo-EM images. Instead of using consecutive patches of a fixed size, cryoSSESeg utilizes density images cropped and masked at the chain level. To deal with the class imbalance in the data sets, multiple loss functions are incorporated. CryoSSESeg is trained and tested using over 1300 chains extracted from experimentally derived cryo-EM density maps. Overall, we obtain residue-level *F*_1_ scores of 0.76 and 0.60 for the detection of helices and β-sheets, respectively, across 33 test cases. A comparison between cryoSSESeg and SSETracer reveals that cryoSSESeg offers robust detection performance, comparable to the best performance of SSETracer, without the need to manually select threshold parameters or generate a skeletonized map using third-party tools. Additionally, cryoSSESeg demonstrates performance comparable to that of Emap2sec.

Although density features of secondary structures, such as α-helices and β-sheets, are distinguishable in cryo-EM density maps at medium resolution, the precise detection of secondary structures from such maps is challenging. One of the challenges arises from the disparity in the density distribution and geometric shapes of secondary structures in the medium-resolution maps. Here, we also examine how the segmentation performance of the classifier is affected by the deviation from the expected shape characteristics of the secondary structures. We observe that depending on the density and shape characteristics (i.e., quality) of the secondary structures, the classifier’s performance may vary within a range of 0.2 in the *F*_1_ score across different bins investigated, which suggests that rigorous evaluation of secondary structure quality in the data set is crucial for comprehending classifier performance.

## Footnotes

a https://www.ebi.ac.uk/pdbe/emdb/.
